# Is it useful to combine sputum cytology and low-dose spiral computed tomography for early detection of lung cancer in formerly asbestos-exposed power industry workers?

**DOI:** 10.1186/1745-6673-9-14

**Published:** 2014-04-17

**Authors:** Michael K Felten, Lars Knoll, Christian Schikowsky, Marco Das, Christian Feldhaus, Kurt G Hering, Alfred Böcking, Thomas Kraus

**Affiliations:** 1 Institute of Occupational and Social Medicine, Medical Faculty, RWTH Aachen University, Aachen, Germany; 2 Department of Diagnostic Radiology, Medical Faculty, RWTH Aachen University, Aachen, Germany; 3 RWE Power AG, Essen, Germany; 4 Department of Diagnostic Radiology, Knappschaftskrankenhaus, Dortmund, Germany; 5 Institute of Cytopathology, Heinrich Heine University, Düsseldorf, Germany; 6 Institute of Pathology, Düren Hospital, Düren, Germany; 7 Department of Radiology, Maastricht University Medical Centre, Maastricht, Netherlands

**Keywords:** Lung cancer, Sputum cytology, Computed tomography, Asbestos

## Abstract

**Background:**

Low-dose spiral computed tomography (LDSCT) in comparison to conventional chest X-ray proved to be a highly sensitive method of diagnosing early stage lung cancer. However, centrally located early stage lung tumours remain a diagnostic challenge. We determined the practicability and efficacy of early detection of lung cancer when combining LDSCT and sputum cytology.

**Methods:**

Of a cohort of 4446 formerly asbestos exposed power industry workers, we examined a subgroup of 187 (4.2%) high risk participants for lung cancer at least once with both LDSCT and sputum cytology. After the examination period the participants were followed-up for more than three years.

**Results:**

The examinations resulted in the diagnosis of lung cancer in 12 participants (6.4%). Six were in clinical stage I. We found 10 non-small cell lung carcinomas and one small cell lung carcinoma. Sputum specimens showed suspicious pathological findings in seven cases and in 11 cases the results of LDSCT indicated malignancies. The overall sensitivity and specificity of sputum cytology was 58.0% and 98% with positive (PPV) and negative (NPV) predictive values of 70% and 97%. For LDSCT we calculated the sensitivity and specificity of 92% and 97%. The PPV and NPV were 65% and 99% respectively.

**Conclusions:**

Our results confirmed that in surveillance programmes a combination of sputum cytology and LDSCT is well feasible and accepted by the participants. Sputum examination alone is not effective enough for the detection of lung cancer, especially at early stage. Even in well- defined risk groups highly exposed to asbestos, we cannot recommend the use of combined LDSCT and sputum cytology examinations as long as no survival benefit has been proved for the combination of both methods. For ensuring low rates of false-positive and false-negative results, programme planners must closely cooperate with experienced medical practitioners and pathologists in a well-functioning interdisciplinary network.

## Background

Lung cancer remains one of the leading types of cancer in the world. In 2008 1.61 million new cases of lung cancer occurred and 1.38 million people died [1]. According to the American Cancer Society, the total number of deaths from all types of lung cancer in the United States for the year 2010 still exceeded that from colon-, breast- and prostate cancers combined [2]. The huge and worldwide public health importance of lung cancer urgently calls for effective and affordable health interventions. The primary objectives should be early detection and treatment in addition to smoking cessation programmes. In the 1970s, the National Cancer Institute of America sponsored three mass-screening programmes using chest X-rays and sputum cytology [3-5]. No differences in mortality were observed between the intervention and control groups [6]. As a result of these and other studies, mass screening for lung cancer was not recommended [7]. In recent studies, where the highly sensitive method of low-dose spiral computed tomography (LDSCT) was used instead of conventional chest X-rays, a higher detection rate of early-stage lung cancers could be demonstrated. But the design of these uncontrolled and non-randomised studies was unsuitable to demonstrate that screening with LDSCT decreases mortality [8]. Consequently, medical guidelines did not recommend lung cancer screening in individuals, when they do not have specific symptoms or an increased disease risk [9,10]. Recent results from the National Lung Screening Trial confirmed a decreasing effect of LDSCT screening on mortality, particularly in those with a high risk of lung cancer [11].

Smoking is the predominant risk factor for lung cancer with a 23 times higher risk in male smokers compared to non-smokers [2,12]. Additional environmental and occupational risk factors are also relevant for the development of lung cancer. Exposure to asbestos dust, especially in combination with cigarette smoke, is a main occupational cause of lung malignancies [13-16]. The health effects of asbestos dust are difficult to measure and control. Some reasons for that are the long latency periods (12–37 years) between exposure and the occurrence of lung cancer and the widespread, unprotected use in the past [17].

In this study, we tested the hypothesis that non-automated sputum cytology may be particularly useful for the diagnosis of centrally located tumours [18,19], whereas LDSCT is more capable of detecting tumours in the peripheral parts of the lung. Following that notion, the best detection rates of early stage lung cancer would be in theory expected by applying both methods. We examined the practicability and efficacy of lung cancer screening in a group of power industry workers, who were heavily exposed to asbestos dust in the past.

## Methods

Our study cohort included 5632 formerly asbestos exposed employees of a major provider of electrical power in Germany. They had been enrolled after confirming by signature some contact with asbestos fibres in the past and giving written consent to participate. Most participants had been involved in general maintenance and repair work in various installations of power generation, including lignite fuelled power plants. Routine occupational tasks involved the handling of asbestos and asbestos containing materials such as the removal of asbestos lagging around the turbines and the spraying of asbestos pulp. The work was carried out without effective personal protective measures or technical ventilation [20].

All medical examinations were done in the framework of a health surveillance programme, which had been set up for the formerly exposed employees with the primary objective to detect newly developing lung cancers in an early stage. Before starting the examinations, we recorded age, tobacco consumption and duration of asbestos exposure for all 5632 participants, assuming that these were the major risk factors for lung cancer development. The required data were collected via self-administered questionnaires. Based on this information, all study participants were allocated to one of three risk groups with an assumed low, medium and high risk of developing lung cancer. For risk stratification, we combined the factors duration of asbestos exposure in years, age and smoking status as follows: duration × [age/50]^3^ × smoking (0.1 = non-smoker, 0.3 = ex-smoker, 1.0 = smoker) [21]. Between March 2002 and December 2006 a total of 4446 (78.9%) participants were examined at least once for asbestos related diseases. Routine examinations included occupational history, medical history, standard interview on current respiratory symptoms, physical examination of the thorax and lung function testing. In contrast to the standard chest X-ray in the other participants, the high risk group was examined annually with LDSCT of the thorax and sputum cytology. Automated sputum-cytometry with certified instruments was not available in Germany at the time sputum samples were obtained for this study. Out of those examined, 3182 participants belonged to the low, 1001 to the medium and 263 to the high risk group. For the analysis presented here, we focused on high risk participants who were not older than 75 years on the reference date of 1 September 2002. This was based on the assumption that all other participants, particularly those in the low risk group and those older than 75 years, would have benefitted significantly less from a combined examination with sputum cytology and LDSCT. That way we ensured for most of our study group an acceptable balance between the likelihood of lung cancer and the possible harms from false-positive sputum findings. Participants, who did not submit a sputum sample to the study laboratory, refused to participate in one of the two screening methods or had no usable results because of other reasons, were excluded from the analysis (n = 76). Our evaluation included 187 high risk participants who fulfilled all admission criteria [21]. They represented 4.2% of those examined (n = 4446) and 3.3% of the total cohort (n = 5632). The research was performed according to the principles of the World Medical Association’s Declaration of Helsinki and obtained approval by the Institutional Review Board of the Medical Faculty of RWTH Aachen University (registration number EK2205).

### Computed tomography scans

**Figure 1:**
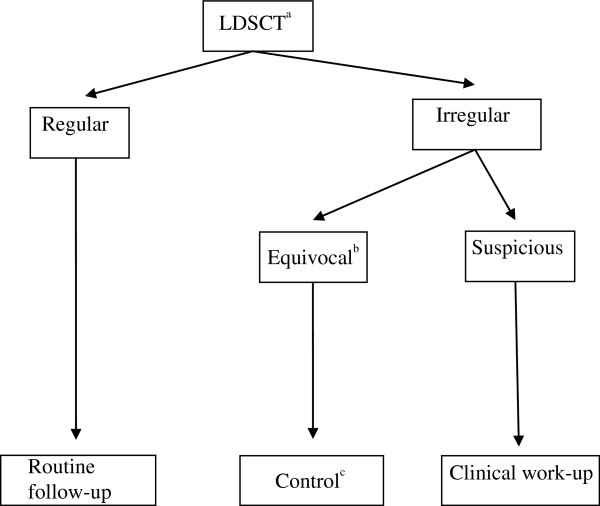
**Flow chart for processing the findings of the low-dose spiral computed tomography of the thorax. **^a^LDSCT = low-dose spiral computed tomography, ^b^Non calcified nodules 6 - ≤10 mm, ^c^3, 6 or 12 month follow-up with LDSCT, depending on size of nodule.

Computed tomography examinations were carried out in a low-dose spiral technique applying 10 mAs_eff._ for persons weighing less than 80 kg, and 20 mAs_eff._ for persons weighing more than 80 kg. No contrast material was administered. All CT-scans were performed with a 16-slice MDCT scanner (SOMATOM Sensation 16, Siemens Medical Solutions, Forchheim, Germany) using the following parameters: 16 × 0.75 mm collimation, rotation time 0.5 sec, table feed/rotation 18 mm. For lung nodule detection a stack of images was reconstructed with 1 mm slice thickness and 0.5 mm increment using a sharp reconstruction kernel (Siemens B50 kernel) and a window centre of −400 HU and a width of 2000 HU. The initial reading was done by an experienced clinical radiologist (MD) at a standard PACS reading workstation (Barco, Philips/Sectra, Stockholm, Sweden). Data were then transferred to a workstation for further evaluation using dedicated lung analysis software (LungCare™, Siemens Medical Solutions, Forchheim, Germany). All CT-scans were routinely re-read by an occupational physician with special experience in the diagnosis of asbestos related diseases (TK). The follow-up algorithm applied for lung nodules has been described elsewhere [21]. In cases with suspicious lung nodules and other pathological findings we recommended a clinical work-up in cooperation with the clinical departments of Internal Medicine, Diagnostic Radiology, Heart and Thorax Surgery, and Radiotherapy (Figure 1).

### Sputum cytology

During medical examination, all participants were instructed in detail on how to generate good sputum samples, how to deposit them in the prepared containers and how to send them to the study laboratory in prepaid envelopes. For sputum collection we used plastic tubes with a screw cap, one for each specimen, containing ca. 50 ml of a fixing solution with 50% ethanol, 2% carbowax 1540 and 0.006% rifampicin [22].

**Figure 2:**
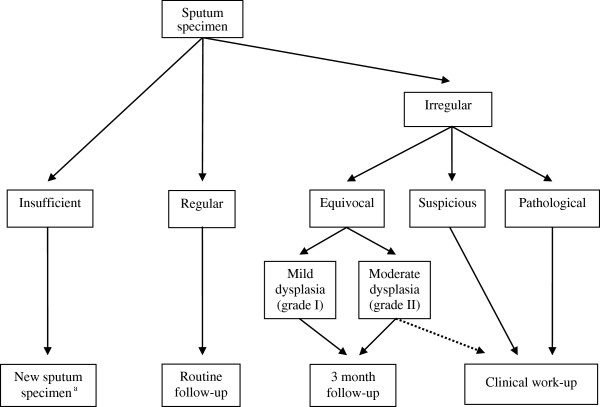
**Flow chart for processing the sputum findings. **^a^Participants were requested to send in a new sputum sample as soon as possible.

The cytological results were categorized in “regular” for normal or inflammatory sputa and “irregular” or “equivocal” for sputa with squamous cell metaplasia in combination with mild or moderate dysplasia (Figure 2). Further categories were “suspicious” for sputa with squamous cell metaplasia and severe dysplasia or very few abnormal cells, and “pathological” for sputa containing malignant cells. In cases of “insufficient” sputum specimens, we requested the participants to send in a new sputum sample as soon as possible. If the assessment was “equivocal” we asked the participants to collect a second set of samples after about three months. In cases of “suspicious” or “pathological” results, we recommended immediate clinical work-up. Appointments with clinicians could be arranged at any time in the framework of the surveillance programme.

A pathological sputum result was considered truly positive if it was confirmed by tissue biopsy or the LDSCT finding clearly indicated lung cancer. Diagnosed lung tumours were classified according to the TNM staging system and their radiological localisation [23]. Tumours located distal to the segmental bronchus were classified as peripheral, all others were considered as centrally located.

### Re-examination

In July 2006 when this study was terminated, 187 participants had been examined at least once with a combination of LDSCT and sputum cytology. Re-examinations after one year and later were possible only in those participants who had their initial examination in or before July 2005. Other reasons for missing re-examination data were refusal to participate again, lack of consent to one of the two investigations and inability to attend because of various reasons such as disease, death or moving away. Of the group of 187 participants, 117 (62%) were re-examined with both methods one year, 66 (35%) two years and 12 (6%) three years after the initial contact. Thus our data pool included the results of 382 parallel examinations with sputum cytology and CT-scan of the thorax.

### Follow-up period

Between August 2006 and the end of 2009 we recorded all new cases of lung cancer in the cohort and analysed their pre diagnosis results during the active study period. That provided us with some indication on the predictive value of LDSCT and sputum cytology. As the study design precluded retrospective analysis of the clinical records backwards from 2009, we evaluated the occupational disease register of the BGETEM as the main secondary source. As an additional source of information, we used death certificates filed before the end of 2009 and indicating the diagnosis of lung cancer.

### Statistical analysis

The sensitivity and specificity of the cytological and radiological investigations was tested against the final diagnosis of lung cancer after three years of follow-up. Furthermore, we calculated the positive (PPV) and negative predictive values (NPV) of sputum cytology and computed tomography. As gold standard for diagnosis, we used the biopsy results obtained in the process of clinical work-up. Calculations of statistical parameters such as sensitivity and specificity were based on the combined results of LDSCT and cytology obtained in the active study period (baseline and re-examination).

## Results

The burden of asbestos exposure and the results for the risk factors age, latency period and cigarette smoke are shown in Table 1. Latency is defined here as the time period between first exposure to asbestos and the date of the initial examination, covering periods of up to 60 years. As some participants were exposed from the beginning of their apprenticeship until retirement age there was a wide range of exposure duration of approximately 35 years. When comparing mean age and latency period, it seems that asbestos exposure started for many participants in their mid-twenties. The mean pack year value was calculated including the results of the active smokers and participants with smoking periods of less than one year.

**Table 1:** Asbestos exposure and other risk factors in the study group (n =187)

	**Mean**	**SD**	**Range**
Age (years)	65.8	5.8	46 – 78
Time since first exposure (years)	40.4	7.8	26 – 60
Duration of asbestos exposure (years)	29.5	7.7	15 – 49
Pack years	44.7	47.9	0 – 118

### New cases of lung cancer during the examination period

In the active study period 12 participants were newly diagnosed with lung cancer (6.4%), 11 of them confirmed with lung tissue biopsy (Table 2). One of the 12 patients (case number four) died suddenly before the diagnosis could be confirmed with lung biopsy. As the patient had clear clinical signs with weight loss of 28 kg within six months together with a fast growing lung nodule reaching 4.7 cm in diameter on CT scan, we considered him also as a confirmed case of progressive lung cancer. Eight lung cancers were diagnosed at the first combined examination (baseline), four at the first re-examination and two in the interval between the baseline examination and the first re-examination. One of the two (case number 13) was diagnosed in an external hospital after symptoms developed. We found ten cases of non-small cell lung carcinoma (NSCLC) and one small cell lung carcinoma (SCLC). Of the 11 participants with biopsy confirmation, six were in clinical stage I, one in stage II, three in stage III and one in stage IV. In half of the 12 confirmed cases the tumours were located in the periphery of the lung.

**Table 2:** Cases of lung cancer among 187 high risk participants with CT scan and sputum examination

**No.**	**Examination**^**a**^	**Location**^**b**^	**Diagnosis**^**c**^	**Histology**^**d**^	**Stage**	**Survival**^**e**^
1	b	per	CT + spu	SQC	IIIa	
2	r	per	CT + spu	SQC	Ia	29
3	b	per	CT	ADO	Ia	
4^g^	b	per	CT			7
5	r	per	CT	LAC	IIIb	23
6	b	per	CT	ADO	Ia	64
7	b	cen	CT + spu	SQC	IV	21
8	b	cen	CT + spu	SQC	Ia	
9	r	cen	CT + spu	SQC	IIb	33
10	b	cen	CT + spu	SQC	Ib	18
11	b	cen	CT	SMC	Ia	28
12	r	cen	spu	SQC	IIIb	72
13	i	cen		SMC	IIb	14
14	i					35
15^h^	f					
16	f			SMC	IIIb	^k^
17	f			SMC	IIb	^k^
18	f				IIIb	^k^

^a^Examination with significant result: b = baseline, r = re-examination, i = interval, f = follow-up.

^b^Location of tumour: per = peripheral, cen = central.

^c^Result indicating diagnosis: CT = low-dose spiral computed tomography, spu = sputum cytology.

^d^Histology of lung carcinoma: SQC = squamous cell, ADO = adeno, LAC = large cell, SMC = small cell.

^e^Survival time after beginning of initial clinical symptoms indicating the disease (months).

^g^Died suddenly 2004 before biopsy could be taken, diagnosis unequivocal clinically and on CT.

^h^Died 2008 with lung cancer after refusing further examinations.

^k^Alive by the end of follow-up.

In patients with missing data the documented histories were incomplete or could not be retrieved in time.

### Sputum cytology

In seven cases with newly diagnosed primary lung cancer, sputum specimens showed suspicious or pathological findings (Table 3). In three other cases with suspicious or pathological sputum specimens, no malignant lung tumours were detected and therefore considered as false positive (false positive rate 1.7%). In the remaining five cases with confirmed lung cancer, the sputum results showed no signs of malignancy and were considered as false negative (false-negative rate 42%). Four of these were peripheral tumours. When comparing the results of sputum cytology with the total number of confirmed lung cancer cases mostly diagnosed with biopsy, we calculated the sensitivity and specificity of cytology with 58% and 98%. The positive (PPV) and negative (NPV) predictive values were 70% and 97%.

**Table 3:** Participants with newly diagnosed lung cancer during the examination period: comparison of sputum cytology with the results of low-dose spiral computed tomography (LDSCT) of the thorax for the whole cohort (n = 187)

	**Lung cancer**	**No lung cancer**^**a**^	**Total**^**b**^
*Sputum cytology*			
Suspicious or pathological^c^	7	3	10
Regular or equivocal^d^	5	172	177
*LDSCT*			
Suspicious	11	6	17
Regular	1	169	170
*Cytology and LDSCT*			
At least one positive	12	8^e^	20
Both negative	0	167	167

^a^Including six lung cancer patients falling ill outside the examination period.

^b^The totals of test results in each group add up to n = 187.

^c^In at least one sputum sample.

^d^In all samples tested.

^e^In one participant, both results were positive.

### Computed tomography

In 11 cases the results of computed tomography indicated malignant lung disease (Table 3). One lung cancer that could not be detected on LDSCT was a patient with a central nodule later diagnosed as squamous cell carcinoma. As the simultaneously done sputum cytology showed a pathological result, that case was counted as a false negative CT result (false negative rate 8.3%). In another six cases with radiological signs of pulmonary or pleural malignant disease the results of clinical work-up did not confirm the radiological diagnosis. Three patients suffered from tuberculoma, an atypical pneumonia and one was diagnosed with dystelectasis of unknown origin. Another two patients showed radiological signs of progressive pleural thickening, consistent with early stage malignant pleural mesothelioma. After explorative surgery including pleural and pulmonary biopsies the final diagnosis of chronic proliferative pleurisy with signs of early lung fibrosis was made. The remaining patient had a large pulmonary nodule, which showed no signs of growth over a period of 46 months and was therefore no longer seen as suspicious (false positive rate 3.4%). For the CT results, we calculated a sensitivity and specificity of 92% and 97%. The PPV and NPV were 65% and 99% respectively. When combining the results of both methods (Table 3), we obtained an overall sensitivity and specificity of 100% and 95%, with a PPV of 60% and a NPV of 100%.

### New cases during follow-up

Of the remaining six patients with lung cancer indicated in Table 2 (cases 13 to 18), two developed their disease in the period between routine examinations (interval) and four after the examination period in August 2006 and the conclusion of follow-up by the end of 2009 (follow-up). None of these patients had pathological or equivocal findings on LDSCT or with sputum cytology in the baseline or re-examinations. The time period between last examination and recorded date of diagnosis lay between 17 and 19 months. In three of the patients we could secure a histological confirmation of the diagnosis lung cancer, one patient died in the year 2008 after refusing further examinations.

## Discussion

The various forms of lung cancer are increasingly common and remain difficult to treat. As long as occupational and environmental causes of lung cancer, such as exposure to asbestos dust and tobacco smoking, cannot be abolished and the chance of recovery mainly depends on the stage of the disease, the early detection of lung cancer is the primary objective of focused health interventions. As the search for an effective biomarker indicating early disease was not successful so far, a combination of sensitive radiological imaging and a filter for detecting high risk individuals seems the most promising approach at present. Our study was based on the simultaneous use of sensitive, state-of-the-art radiological imaging and elaborate sputum cytology applied to a highly selected group of individuals. In theory, the combined use of imaging and cytology should help to further reduce the “blind spot” of radiological imaging regarding the early detection of central lung tumours. Other complementary screening strategies for lung cancer are under investigation, but they are either too invasive as a first-step screening method, such as fluorescence bronchoscopy, or their practical value has not been proven yet, such as exhaled breath analysis [24,25]. Our hypothesis was that parallel cytology could detect invisible, early stage central tumours or quickly confirm the malignant nature of tumours with a difficult location for taking biopsies. However, our highly selected but small study group together with the limited follow-up period were serious limitations for testing that hypothesis. It was possible that we might have missed some patients with newly diagnosed lung cancer, who were alive and were not reported to the BGETEM as suspect cases of occupational lung cancer. We would also have missed deceased lung cancer patients with a false or misleading diagnosis on their death certificates. From a practical point of view, the secondary objective of establishing a novel approach under routine working conditions appeared almost as important. While sputum cytology in combination with other screening methods has been tested before, there are no recent reports on larger scale use together with LDSCT in asbestos exposed individuals [25-27]. Our evaluation should therefore also be seen as a contribution to the debate, whether or not a formerly asbestos exposed participant of an early detection programme for lung cancer is willing to undergo additional, often inconvenient sputum testing. Large scale health programmes, even in individuals with an increased risk of disease, would be open for criticism when launched without convincing evidence of both, increased survival and reduced all-cause mortality. Due to scope and design of the study, we could not assess the overall effectiveness of lung cancer screening in asbestos exposed individuals. However, our results provide good indication of the value of a combined approach using CT-scans and sputum cytology.

When focussing on the occupational risk of lung cancer caused by the exposure to airborne asbestos, the identification of high-risk individuals is complicated by the formerly widespread, unprotected use of asbestos and the extremely long latency periods between exposure and disease. In our effort to form a high-risk group, who would benefit most from lung cancer screening, we linked the data on asbestos exposure with age and smoking habits, which resulted in a highly selected group of 4.2% of the total [21]. We could therefore assume with some certainty that the participants most at risk of developing lung cancer were included in the study group. However, it was also clear that the exposure data based on self-administered questionnaires might have been affected by a potential recall bias, especially in those who had started work several decades before. The data on cumulative exposure to asbestos and tobacco smoke (Table 1) should therefore be seen in the light of the long time periods since first exposure, stretching in some over an entire working life, and interpreted with caution.

### Examination methods

In large-scale surveillance programmes, examination methods must be easy to handle and fit into tight working schedules. The methods should be standardized, not invasive and easily tolerated by the participants. The values for sensitivity and specificity should be well over 90%. In the light of these requirements, LDSCT seems to be an examination method, which is well suited for health surveillance. CT-scans can be performed within a few seconds, not causing any physical inconvenience or requiring a specific activity by the patient. The method of sputum collection at home is not invasive and is also a well-accepted procedure. Nevertheless, this technique is little standardized and the collection of sputum samples at home precludes technical guidance by an experienced person. It was therefore impractical to use induced sputum samples which would have required some technical support for the saline aerosol inhalation as usually only available at hospital. It was however clear that sputum induction would have allowed better quality samples and more valid results. In the context of our study, the approach of routine sputum sampling still proved to be a well feasible and acceptable surveillance method.

### False-positive results

For participants with false positive results, the examination with LDSCT or sputum cytology had significant consequences. They usually underwent invasive and actually unnecessary examinations such as lung biopsy or thoracotomy with the risk of adverse effects and potentially even affecting the survival time of the individual. Health surveillance programmes for the early detection of lung cancer with high rates of false positive findings would be ineffective and difficult to justify. Humphrey et al. described recent LDSCT studies in which the false positive rates ranged from 5% to 50% of prevalence and 3% to 12% of incidence [8]. While in our study the false positive rates were minimal in comparison (1.7% with 3 of 175 sputum examinations and 3.4% with 6 of 175 LDSCT examinations), the positive predictive values of 70% and 65% respectively, and the resulting precision of sputum cytology and LDSCT were unsatisfactory. When combining the results of both methods, the resulting PPV of 60% was still lower. This was accompanied by a corresponding increase of sensitivity to 100%. Eight participants had to undergo actually unnecessary further investigations, which were however limited to sputum and CT controls after three months in the three participants with irregular sputum results. When defining as “false-positive” only those results leading to invasive clinical work up with negative outcome, we had no false positive sputum results.

Another important aspect when considering the consequences of false positive screening results was the fact that individuals informed on the results will usually experience an extended period of anxiety and concern before the results were confirmed false. That was also the case with one of our participants, who was admitted to a psychiatric institution for treatment of depression after being informed that a lung nodule suspicious of lung cancer had been found. It seems therefore essential that large scale early detection programmes for life threatening diseases such as lung cancer are carried out with the support of a network of specialists also including psychologists and psychiatrists, who can attend to individual patients.

### Sputum findings

In a review of 16 representative studies to assess the sensitivity of sputum cytology, values ranging from 42% to 97% were calculated, depending on the location of the lesions [19]. However, the variety of methods made it difficult to assess the validity of these data. In our study, the calculated sensitivity of sputum cytology was with 58% low in comparison with the value for LDSCT of 92%. We considered the finding that one centrally located lung tumour invisible on LDSCT was detected by sputum cytology (in stage IIIb) as at least demonstrating the potential benefit of the method in a surveillance effort.

One of the false negative cases was equivocal for sputa with squamous cell metaplasia with moderate dysplasia (grade II). For such cases, we recommended a control sputum examination after three months (Figure 2). If immediate clinical work-up (Figure 2, dotted line) would be done, the rate of false-negative findings could be reduced and sensitivity improved. However, the positive predictive value would also have decreased from 70% to 53.8%, resulting in a reduced precision of sputum cytology and more false-positive results. To identify malignant cells by means of sputum cytology requires experienced personnel with a high degree of expertise. Sputum analysis using DNA image cytometry as an automated computer-assisted method to detect malignant cells may be an alternative, which is more suitable for widespread routine use. Sputum cytometry alone or better in combination with sputum cytology might increase the sensitivity of sputum examinations by 70 to 90% [28-30].

### Location of lung tumours

The importance of tumour location on the sensitivity of sputum cytology has been described in previous studies [19,31]. Most of them showed decreased sensitivity for peripherally located masses, others could not find any difference. Our results seemed to indicate that centrally located tumours were diagnosed more often by sputum cytology (83%) than peripheral tumours (33%). One plausible explanation might be the closer contact of central tumours to the bronchogenic system. It may well be that in cases with a centrally located tumour the closer contact to the bronchogenic system could compensate for the higher risk of a false negative result of the CT scan. That risk is further increased by the standard technique of CT without contrast material.

### Follow-up

Participants with normal examination results, who were diagnosed with lung cancer during follow-up, might be seen as “false negative” cases. However, the delay between examination and diagnosis let it appear unlikely that any signs of a developing carcinoma could have been picked up by CT scan or sputum cytology. The fact that four cases among a group of about 200 over a period of more than three years corresponded roughly with the expected true incidence may also indicate that specific symptoms and detectable signs of disease developed only after the examinations. As none of the participants with false positive results developed disease during follow-up, we can assume that they were truly false positive cases and the applied algorithm was effective in ruling out early stage lung disease.

## Conclusions

Our results confirmed that (1) in surveillance programmes a combination of sputum cytology and LDSCT is well feasible and accepted by the participants. (2) Sputum examination alone is not effective for the detection of early stage lung cancer. (3) In high risk individuals, the combination of LDSCT scans and sputum cytology is probably more effective in detecting early lung cancer than LDSCT alone. However, the combination of both methods cannot be generally recommended as long as there is no evidence of a significant reduction of mortality and an improvement in overall survival. (4) For keeping false-positive and false-negative findings low, it is essential to ensure close cooperation with experienced clinicians and pathologists in a well-established interdisciplinary network.

## Competing interests

The authors declare that they have no competing interests. This survey was supported financially by the Institution for Statutory Accident Insurance and Prevention in the Energy, Textile, Electrical and Media Industry (BGETEM, grant number 360057) and RWE Power AG (grant number 370221) with unrestricted grants to the Medical Faculty, RWTH Aachen University.

## Authors’ contributions

MKF and LK organized the cohort, examined most of the participants, managed the survey data and drafted the manuscript. TK, KGH and CF conceived the survey and planned the building of the cohort. TK and AB managed the collection of the sputum samples, AB organised and supervised their examination and interpreted the results. MD and TK read the CT-scans and interpreted the results. LK and CS did the statistical analysis of the survey data. All authors read and approved the final manuscript.
